# Intestinal Taste Receptor Expression and Its Implications for Health: An Integrative Analysis in Female Rats after Chronic Insect Supplementation

**DOI:** 10.1021/acs.jafc.4c02408

**Published:** 2024-06-10

**Authors:** Helena Segú, Florijan Jalševac, Mònica Lores, Raúl Beltrán-Debón, Ximena Terra, Montserrat Pinent, Anna Ardévol, Esther Rodríguez-Gallego, Maria Teresa Blay

**Affiliations:** MoBioFood Research Group, Departament de Bioquímica i Biotecnologia, Universitat Rovira i Virgili, c/Marcel·lí Domingo n°1, 43007 Tarragona, Spain

**Keywords:** taste receptors, insect, umami, bitter, intestinal function, integrative analysis, inflammation

## Abstract

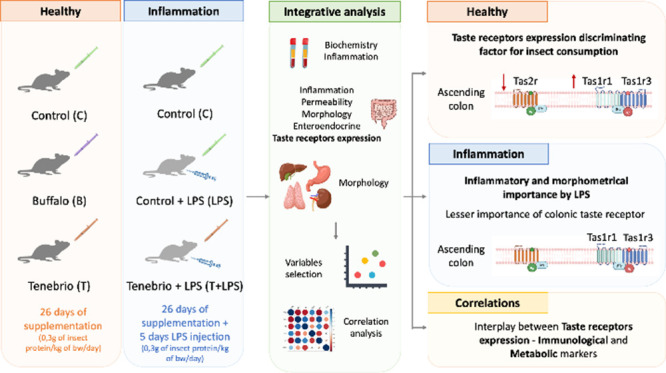

Taste receptors are found in the gastrointestinal tract, where they are susceptible to dietary modulation, a key point that is crucial for diet-related responses. Insects are sustainable and good-quality protein sources. This study analyzed the impact of insect consumption on the modulation of taste receptor expression across various segments of the rat intestine under healthy or inflammatory conditions. Female Wistar rats were supplemented with *Tenebrio molitor* (T) or *Alphitobius diaperinus* (B), alongside a control group (C), over 21 days under healthy or LPS-induced inflammation. The present study reveals, for the first time, that insect consumption modulates taste receptor gene expression, mainly in the ascending colon. This modulation was not found under inflammation. Integrative analysis revealed colonic Tas1r1 as a key discriminator for insect consumption (*C* = 1.04 ± 0.32, *T* = 1.78 ± 0.72, *B* = 1.99 ± 0.82, *p*-value <0.05 and 0.01, respectively). Additionally, correlation analysis showed the interplay between intestinal taste receptors and metabolic and inflammatory responses. These findings underscore how insect consumption modulates taste receptors, influencing intestinal function and broader physiological mechanisms.

## Introduction

Taste receptors are predominantly located in taste buds in various places throughout the oral cavity, where they play a crucial role in recognizing exogenous compounds in food and beverages and other ingested substances. The signals from these receptors are transmitted via afferent gustatory nerves to the brain structures involved in central taste processing.^1^ However, these receptors are not limited to the oral cavity as they can also be found in extra-oral tissues and organs throughout the body, from the brain and skin to the reproductive system and gastrointestinal tract.^2^

Taste receptors are responsible for identifying diverse tastes, including sweet, bitter, salty, sour, and umami. While salty and sour tastes are recognized through ion channels,^3^ umami, sweet, and bitter receptors belong to the G-protein-coupled receptor (GPCR) superfamily and are divided into two types: TAS1R, which are responsible for sweet and umami tastes, and TAS2R, which identify bitter taste. Among type-1 receptors, Tas1r1, Tas1r2, and Tas1r3 operate as heterodimeric duets. Specifically, the Tas1r2-Tas1r3 heterodimer is responsible for recognizing sweetness while L-amino acids and ribonucleotides interact with Tas1r1-Tas1r3, which constitutes the taste sensation known as umami.^4,5^ With regard to bitter taste receptors, 26 and 37 distinct subtypes have been reported in humans and rats, respectively. However, data regarding the presence of these receptors along the intestine in animal models are still limited. Notably, Tas2r108, Tas2r119, Tas2r138, and Tas2r143 are among the subtypes that exhibit the highest expression levels of all bitter receptors in rats.^6^ Moreover, each subtype is susceptible to activation by specific bitter compounds or a broad spectrum of them.^7^ However, some of these bitter receptors, including Tas2r108, Tas2r119, Tas2r138, Tas2r139, and Tas2r143, have also been shown to be sensitive to certain peptides and amino acids.^8−12^ This study focuses on the modulation of TAS1R and the TAS2R expression mentioned above along the intestine.

The abundance and tissue distribution of taste receptors, especially in the gastrointestinal tract, raises the prospect of their involvement in physiological functions.^13^ Since the intestine serves as the primary organ for food digestion, a process that occurs over a relatively extended period, chemosensory receptors in the gastrointestinal wall are subjected to prolonged exposure to agonists and antagonists present in ingested food.^14−17^ These components can interact with various receptors, binding and activating them, sending to diverse parts of the organism this information through different kinds of signaling molecules. The localization of taste receptors in the enteroendocrine cells plays a role in regulating the secretion of enteroendocrine peptides, including ghrelin, GLP-1, PYY, and CCK.^4,18−20^ Additionally, several studies have proposed a potential role for taste receptors in the immune function in view of their placement on Goblet and Paneth cells.^18,21^ Some researchers have also demonstrated the ability of dietary components to exert a profound effect on the genetic expression of these receptors in the intestine.^2,4,16,22^ In this context, exploring how a diet or dietary compound interacts with taste receptors becomes crucial for discerning any potential beneficial or detrimental effects.

Protein is essential for human nutrition as it plays an important role in the formation and repair of tissues and the proper functioning of enzymes and hormones.^23,24^ Ensuring sufficient protein intake is crucial for maintaining robust immune function, supporting optimal muscle development, and protecting overall health.^25^ Abundant evidence strongly supports the postulation that insects are a source of high-quality protein,^26,27^ and are considered sustainable sources of protein that require fewer land and water resources than conventional livestock production.^27,28^ Based on evaluated studies on the microbiological risk of zoonosis, heavy metal contamination, and allergenicity, the European Food Safety Authority (EFSA) has given a favorable opinion on four insects as novel foods, including two mealworms from different beetle species, *Tenebrio molitor* and *Alphitobius diaperinus*, which serve as the focus of study in this article.^29−32^ These two insect species, belong to the Tenebrionidae family of the order Coleoptera, highlighting their taxonomic proximity and the nutritional composition at the larval stage is similar.^33^*T. molitor* has been used across various sectors and in *in vivo* studies and it is considered one of the most promising insect proteins in the food and feed industries.^34^ However, *A. diaperinus* has been less commonly employed, necessitating further studies on their health effects.^35^ In this context, some studies have highlighted the bioactive properties of insect peptides, while demonstrating their potential as antihypertensive, anti-inflammatory, antidiabetic, or antioxidant agents.^36^ Nevertheless, new studies that provide evidence of the health advantages of insect consumption, as well as the precise mechanism of action, are still needed. In this sense, prior findings from our research group indicated that chronic low-dose supplementation of *A. diaperinus* in rats decreased local ghrelin levels in the small intestine and increased food intake.^33^ Moreover, our research team has conducted several studies to investigate the impact of *A. diaperinus* and *T. molitor* on intestinal immune function and morphology, under both healthy and inflammatory conditions. Our findings demonstrated healthy responses in terms of systemic and intestinal inflammation, allergenic response, and intestinal morphology in rats after chronic insect supplementation in both conditions.^37^ However, there is still a lack of evidence for the role of extra-oral taste receptors after insect consumption and its potential implications for intestinal health. Considering the presence of 5-ribonucleotides and several amino acids or peptides specific to insects, these are expected to interact with type I and some type II taste receptors in the intestinal tract, thereby adding a novel dimension to our exploration of the broader impact of insect consumption on intestinal health.^38^

Given the evidence supporting the potential benefits of insect consumption and the recognized importance of taste receptors in maintaining overall health, our study aimed to explore how dietary components modulate intestinal taste receptors across different health conditions. Specifically, we investigated the broader impact of chronic daily insect supplementation on taste receptor expression in the rat intestine, encompassing both physiological and inflammatory conditions, with the main aim of elucidating the complex interplay between dietary interventions and organism responses. Through an integrative analysis approach, we explored the modulation of the expression of intestinal taste receptors by insect protein and their potential role in distinguishing between groups receiving insect supplementation and those that do not. Furthermore, in this study, we used an LPS-induced inflammatory model, a well-established experimental approach for studying intestinal dysfunction and systemic alterations, including increased intestinal permeability and exacerbation of inflammation.^39,40^ Through this model, we aimed to gain valuable insights into the mechanisms underlying taste receptor modulation and its relationship to overall health parameters during inflammatory conditions. Additionally, we evaluated the effect of species-specific supplementation, evaluating the most commonly studied mealworm (*T. molitor*) and another less-used larvae (*A. diaperinus*).

## Materials and Methods

### Chemicals

Lipopolysaccharide (LPS) from *Escherichia coli* O111:B4 (impurities ≤3.00% protein) (Merck Lifesciences, Madrid, Spain; Cat No:4357765). Standard Teklad diet (Envigo++, Barcelona, Spain; Cat No: Teklad 2014). *Tenebrio molitor* flour (Iberinsect, S.L; Reus, Spain), *Alphitobius diaperinus* flour (Protifarm NV, Ermelo, Gelderland, The Netherlands). The nutritional compositions of these two insect flours are described in Table 1. TRIzol reagent (Thermo Fisher Scientific, Waltham, MA, USA). Capacity cDNA Reverse Transcription kit (Applied Biosystems, Madrid, Spain), Specific TaqMan probes (Thermo Fisher Scientific, Madrid, Spain).

**Table 1:** Nutritional Composition of the Administered Treatments Measured on Dry Matter (Values per 100 g of Insect Flour)

**composition**	**A. diaperinus**	**T. molitor**
energy (kJ)	2550	2604
protein (g)	56.31	56.1
total lipids (g)	18.82	26.31
starch (g)	1.30	3.34
fiber (g)	7.44	7.78

### Experimental Design

**Figure 1:**
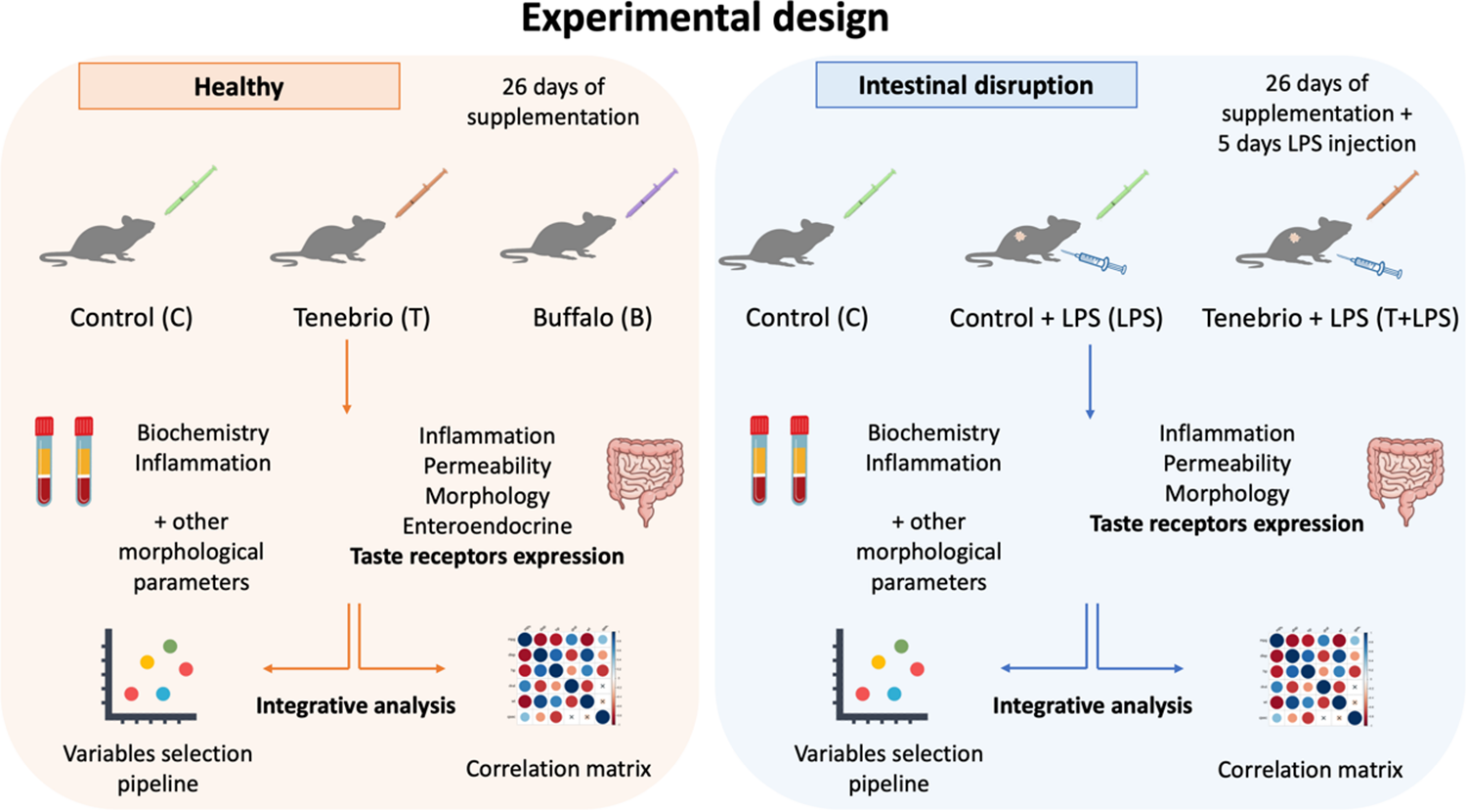
Experimental design.

Forty female rats were acclimated for 14 days under standard conditions (22 °C with a 12 h light-dark cycle). During this period, they had *ad libitum* access to water and were fed a standard Teklad diet (Envigo++, Barcelona, Spain; Cat No: Teklad 2014). After this period of adaptation, the rats were divided into five experimental groups (8 rats per group), categorized into healthy and inflammatory conditions (Figure 1). The healthy condition included a control group (Control) fed a standard diet, a group supplemented with *Tenebrio molitor* flour (*Tenebrio*) (300 mg/kg bw/day), and a group supplemented with *Alphitobius diaperinus* flour (Buffalo) (300 mg/kg bw/day). On the other hand, the inflammatory condition included a control group receiving 5 days of intraperitoneal lipopolysaccharide (LPS) injections at 0.5 mg/kg of body weight (LPS group), and a group receiving both *Tenebrio molitor* flour and LPS (*Tenebrio* + LPS group). The intervention lasted for 26 days, and additional details of the experimental design have been described in a previous article.^33^

After sacrifice, blood was centrifugated to obtain plasma, while tissue samples, including various intestinal segments (duodenum, jejunum, ileum, ascending colon, and descending colon), were rapidly removed, weighed, and frozen in liquid nitrogen before storage at −80 °C until further analysis. After thorough cleaning and removal of fat, samples from the duodenum, jejunum, ileum, ascending colon, and descending colon were demarcated according to the intestinal segment division described by Vdoviaková *et al.*(41) All procedures were approved by the GENCAT Animal Experimentation Committee (number 11701).

### Gene Expression of Intestinal Taste Receptors

Total RNA was extracted from the entire tubular tissue from each segment of the intestine using TRIzol reagent (Thermo Fisher Scientific, Waltham, MA, USA) and following the manufacturer’s instructions. Complementary DNA (cDNA) was obtained using a High-Capacity cDNA Reverse Transcription kit (Applied Biosystems, Madrid, Spain), as described in a previous study.^42^ Quantitative PCR amplification was performed from a total of 40 ng/μL of cDNA using TaqMan Universal PCR Master Mix (Applied Biosystems, Madrid, Spain) and specific TaqMan probes for PPIA (cyclophilin A) (Rn00690933_m1), Tas1r1 (Rn01516038_m1), Tas1r2 (Rn01515494_m1), Tas1r3 (Rn00590759_g1), Tas2r108 (Rn02396427_s1), Tas2r119 (Rn00576950_s1), Tas2r138 (Rn02396417_s1), Tas2r139 (Rn04218919_s1) and Tas2r143 (Rn02585801_s1) genes from Thermo Fisher Scientific. The relative expression of each gene was compared with the control group using the 2^–ΔΔCt^ method^43^ and with the cyclophilin gene as the endogenous control gene.

## Statistical Analysis

### Univariate Analysis

Analyses were performed with XLSTAT 2022 (Addinsoft, USA). A normality test for each group was conducted using the Shapiro-Wilk test. The relative expression of taste receptors is presented in box and whiskers plots, where boxes represent the median and interquartile range and whiskers go down to the smallest value and up to the largest, encompassing the full range of the data. Moreover, the written values are expressed as mean ± standard deviation (SD). Pairwise comparisons for statistical differences were conducted using the Mann–Whitney test. *p*-values <0.05 were considered statistically significant. These analyses were conducted in two separate conditions: healthy which included Control, Buffalo, and *Tenebrio* groups; and inflammation condition which included Control, LPS, and *Tenebrio* + LPS. Additionally, the Fold Change of each gene expression was calculated as A/B, with A representing the gene expression mean of the *Tenebrio* or Buffalo group and B representing the gene expression mean of the control group when the analysis included the healthy rats. When focused on animal groups under inflammatory situations, the Fold Change was calculated by taking the gene expression mean of the Control or *Tenebrio* + LPS group as A and the gene expression mean of the LPS group as B.

### Integrative Analysis and Variable Selection Pipeline

All data processing, integration, variable selection pipeline, and statistical analysis described in this section were performed using RStudio version 2023.03.1 Build 446 (2009–2023 Posit Software, PBC).

Data encompassing morphometric, biochemical, immunological, and intestinal permeability analysis in this study have previously been collected, analyzed, and used for research purposes.^37^ These data include tissue weights, intestinal lengths, and biochemical analyses (including glucose, triglycerides, urea, cholesterol, creatinine, and β-hydroxybutyrate). Inflammatory and allergenic parameters in both plasma and intestine (TNF-α, IL-1β, IL-10, secretory IgA, myeloperoxidase (MPO) activity, IgE, histamine, and the relative gene expression of IgA and IL-1β) are as well as intestinal permeability. Moreover, results on the secretion of the enterohormones (GLP-1, ghrelin, and insulin), also included in this analysis, have already been published.^33^ Additionally, this integral analysis also included the relative gene expression of intestinal taste receptors, which are unique variables to this study and have not been previously used.

All raw data, with medians calculated for missing values and redundant variables removed to reduce data dimensionality and collinearity, were preprocessed. The resulting integrated data consisted of four metabolic variables, six biochemical variables, nine general and twenty-seven intestinal morphometric variables, 12 inflammatory variables, and thirty-one TASR gene expressions. The data were centered and scaled using the “ScaleData” function.

With all the variables, the 'RunPCA' function was employed to conduct principal component analysis (PCA) and determine if samples formed groups or clusters, or if any animal was an outlier that needed exclusion in later steps.

The variables were further analyzed in a multivariate approach by our variable selection pipeline, which takes the consensus of three machine learning methods: Elastic Net, Partial Least Squares Discriminant Analysis (PLS-DA), and Random Forest Analysis (RF). Elastic Net is useful for dealing with data sets containing a large number of features, some of which are highly correlated. PLS-DA is a versatile statistical method for classifying and discriminating in high-dimensional data sets, which makes it well suited for our objective to distinguish between different experimental groups based on the variables analyzed. RF is a powerful, flexible algorithm from the family of tree-based models that can be used for both classification and regression.

Our experimental design encompassed two scenarios: one involving healthy rats and comprising the control, *Tenebrio*, and Buffalo groups, and the other involving rats with induced inflammation and comprising the LPS and *Tenebrio* + LPS groups. We therefore conducted two separate analyses by applying the algorithms to each scenario and comparing (1) the control group with the Buffalo or *Tenebrio* group and (2) the LPS group with the control or *Tenebrio* + LPS group. With this approach, we were able to examine the effects of chronic insect supplementation in both a homeostatic status and an LPS-induced inflammation situation.

Nonzero coefficients from a subset of variables selected from the Elastic Net model, variable importance for projection (VIP) coefficients from the PLS-DA model, and mean decrease Gini values from the RF model were used as measures of variable importance.

Each method generated a set of scores that reflected the importance of variables in relation to the aim of distinguishing between groups. These scores were treated as individual “scores” for each variable within each method. We then calculated a “total score” for each variable by summing its “scores” generated by the three methods. This “total score” serves as an indicator of the overall importance of the variable within the context of the study. The variable with the highest “total score” was considered the most important one in consensus. In other words, the variable that was selected by most methods and achieved the highest score in each of the four methods was identified as the variable of greatest significance in this study. With these results, an integrative analysis that ranked all variables based on their importance or obtained score was conducted. To refine this ranking and identify the most critical variables for distinguishing among our study groups, we applied the Kneedle algorithm. This algorithm identifies the knee (or elbow) point on the curve formed by the sorted importance scores of the variables while demarcating the most influential variables. This selection method enabled us to focus our subsequent analyses on those variables that displayed the greatest discriminative power between the groups, thus ensuring a more targeted and effective investigation.

A Venn Diagram was created to integrate the two analyses in each case: (1) control versus Buffalo with control versus *Tenebrio*; and (2) LPS versus control with LPS versus *Tenebrio* + LPS. This diagram enabled us to determine whether any variable that is crucial in the separation of two groups is also significant in distinguishing one group from a third group. In this context, variables present in the intersection of two ellipses are common to both comparisons. These were then visualized in a Fold Change heatmap that describes how much the variable changes between two conditions, as well as the direction of the change.

Finally, a heatmap based on Spearman correlations of taste receptor expression was conducted to explore potential associations between the relative expressions of taste receptors and the other parameters studied in the experiment.

## Results

### Insect Consumption Primarily Modulates Taste Receptor Gene Expression in the Ascending Colon

**Figure 2:**
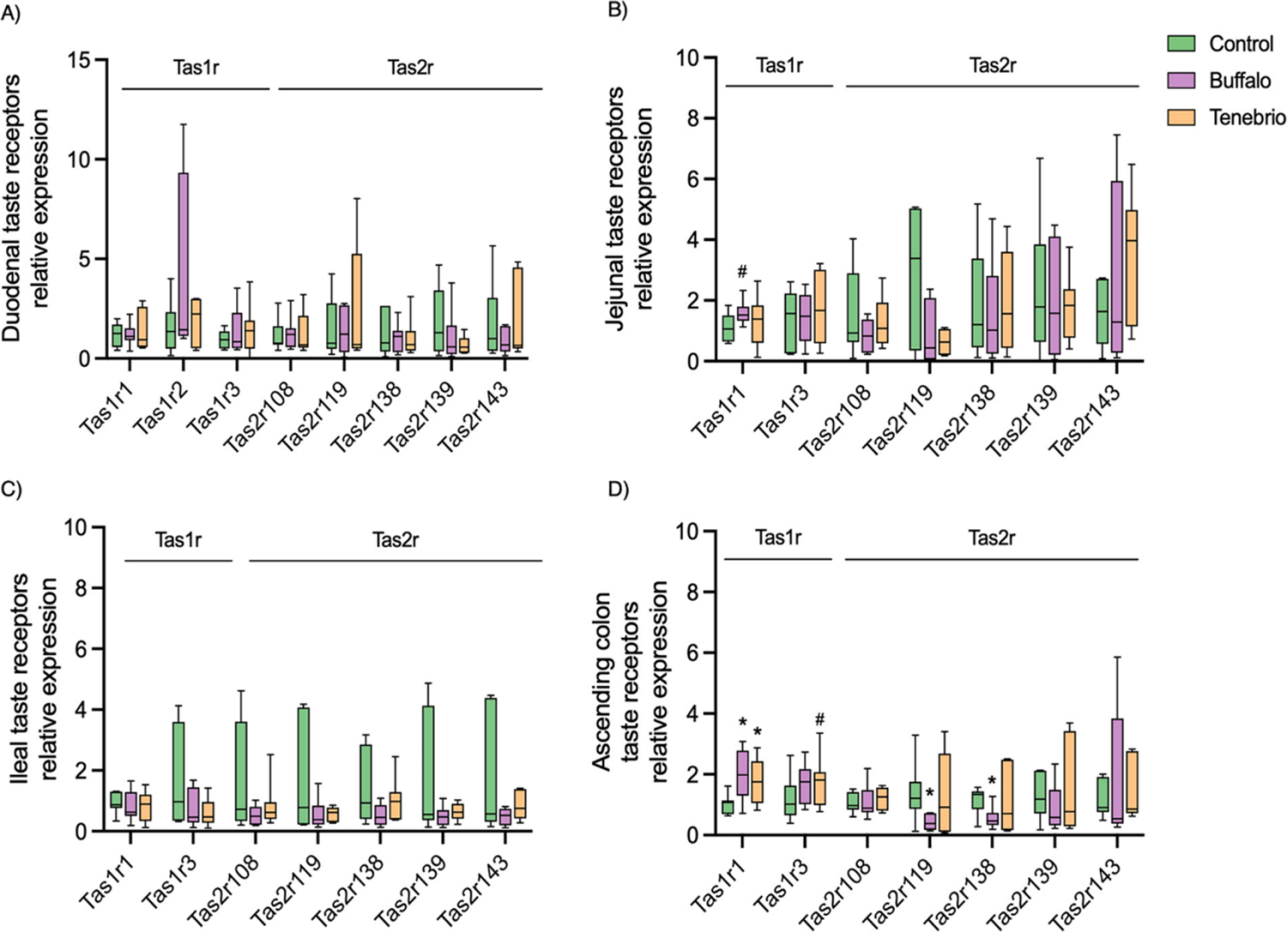
Relative expression of intestinal taste recepeptors. Animals supplemented with Buffalo for 21 days are represented in purple, those receiving *Tenebrio molitor* in orange, and the control group that received water as a vehicle are depicted in green. The data are presented in a box and whiskers plot. Boxes represent the median and interquartile range, while whiskers extend from the smallest to the largest values, encapsulating the full range of the data. Mann–Whitney analysis were used to compare each insect-supplemented groups with the control group (*n* = 7–8/group). * indicates *p*-value <0.05 and # indicates 0.05 < *p*-value <0.1 compared to control group.

To address the modulation of the abundances of the main described bitter taste receptors located at the different intestinal locations^6^ and umami receptors, we work with the quantification of their mRNA. It allows us to run a quantitative screen of eight of them, as indicative of the potential proteins to be located in the membrane to act as truly receptors for their respective ligands. In the small intestine of healthy female rats, the consumption of both insect species induced no significant changes in the relative expression of the taste receptors assayed when compared with the control group (Figure 2A–C). Taste receptors in the duodenum (Figure 2A) were not modified by initially hydrolyzed proteins from the stomach. However, the expression of umami taste receptors (Tas1r1 and Tas1r3) in the ascending colon increased when the rats were supplemented with either Buffalo or *Tenebrio* (Figure 2D). Note that at this location most protein digestion was completed. A tendency for the Tas1r1 profile to increase was also found in the jejunum when the rats were administered the Buffalo supplement (Figure 2B). Finally, with regard to bitter taste receptors, the Buffalo-supplemented group exhibited significantly lower relative expression levels of Tas2r119 and Tas2r138 in the ascending colon than did the control group (Figure 2D).

In the descending colon, we measured only Tas1r1 and Tas1r3 receptors. The gene expression of Tas1r1 in the groups under study was similar (control: 1.12 ± 0.44; Buffalo: 1.31 ± 0.73; *Tenebrio*: 1.36 ± 0.60; *p* > 0.05). The same pattern was observed with regard to Tas1r3 expression, levels of which between groups were similar (control: 1.06 ± 0.37; Buffalo: 1.31 ± 1.15; *Tenebrio*: 1.73 ± 0.96; *p* > 0.05).

### Tas1R1 in the Ascending Colon Is a Discriminating Factor for Insect Consumption

To analyze the importance of taste receptors in the intestine for overall intestinal health, we used machine learning algorithms to integrate and identify which variables among the ninety-seven we analyzed distinguished between groups most effectively. We conducted this analysis under healthy conditions by pairing each insect species with the control group.

**Figure 3:**
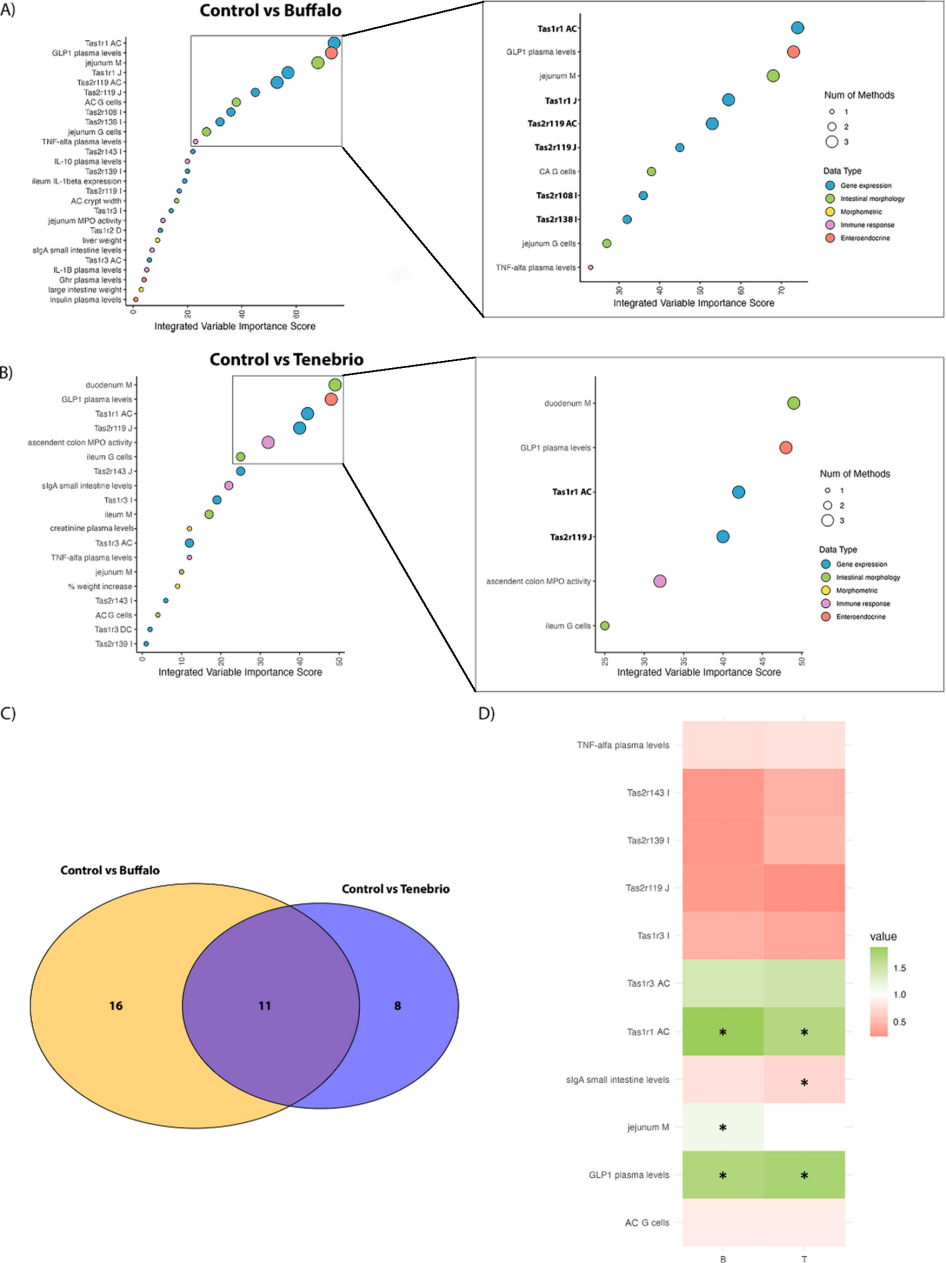
Principal variables distinguishing between the control group and insect-supplemented groups. (A and B) integrate the analysis of selected variables using machine learning algorithms, ranking them to distinguish between Buffalo and Control or *Tenebrio* and Control groups, respectively. (C) Venn Diagram derived from the integrative analysis. (D) Fold change Heatmap for the 11 variables that are common in the two comparisons. Green color indicates the gene expression upregulation or higher levels of the variable, while red signifies downregulation or reduced levels, compared to control. * indicates *p*-value <0.05. Abbreviations utilized include D (duodenum), J (jejunum), I (ileum), AC (ascending colon), DC (descending colon), G (goblet cells) and M (total absorptive area).

Elastic Net, PLS-DA, and Random Forest analyses identified twenty-seven key variables that distinguished between the control and Buffalo groups. These variables were then integrated, ranked, and visually presented in the Dot Plot shown in Figure 2A. Among these variables, a striking representation of taste receptor expression across various segments of the intestine was observed. After applying the Kneedle algorithm to select a subset of the most important of these variables, we selected a total of 11. Among these top variables identified as highly discriminative between groups, the expression levels of certain taste receptors consistently stand out. Specifically, the relative expression levels of Tas1r1 in the ascending colon (Tas1r1 CA) and the jejunum (Tas1r1 J) were consistently identified by all three machine learning algorithms as key variables that effectively distinguish between the control and Buffalo groups (Figure 3A). The expression of bitter taste receptor Tas2r119 in both the jejunum and ascending colon was also identified as a significant variable by two and three methods, respectively.

When data from the control and *Tenebrio* groups were also subjected to Elastic Net, PLS-DA, and Random Forest analyses, 19 variables were selected (Figure 3B). The key discriminative factors again consistently included the relative expressions of taste receptors between these two groups. More specifically, the expression of Tas1r1 in the ascending colon and the expression of Tas2r119 in the jejunum once more emerged as pivotal variables consistently selected by all three methods for differentiation in this comparison, thereby echoing their significance from the previous analysis. To summarize these analyses, the gene expression of Tas1r1 in the ascending colon and that of Tas2r119 in the jejunum indicate that these taste receptors are highly modulated by chronic insect supplementation.

The Venn Diagram (Figure 3C) illustrates the number of variables that distinguish the Buffalo or *Tenebrio* group from the control group and the number of common variables in both insect treatments (represented in the intersection). Of the 35 variables that are able to distinguish the insect groups from the control group, 11 were identified as common discriminators. These variables are showcased in the Fold Change Heatmap (Figure 3D), which demonstrates the extent of change for each variable comparing Buffalo or *Tenebrio* supplemented groups and the control group. The color of each variable indicated that all selected variables exhibit modifications (asterisks indicate those that are statistically significant) in the same direction with insect supplementation, with six of these corresponding to the relative expressions of intestinal taste receptors. Interestingly, general observations across both insect groups were an increase in the expression of colonic umami taste receptors (Tas1r1 and Tas1r3) and a consistent downregulation of some bitter taste receptor expression in the jejunum or ileum. This suggests that insect supplementation could modulate intestinal taste receptors by enhancing colonic umami receptor expression and suppressing some bitter receptor expression. Note also that the expression of Tas1r1 in the ascending colon serves as a pivotal receptor for insect consumption, underscoring its crucial role in discriminating between groups that consume insects and control groups. This significance is evident in both multivariate discriminative analysis and the statistically significant differences observed in univariate analysis between the insect-treated groups and the control group.

### Modulation of the Relative Expression of Intestinal Taste Receptors under a Proinflammatory Stimulus Is Limited

**Figure 4:**
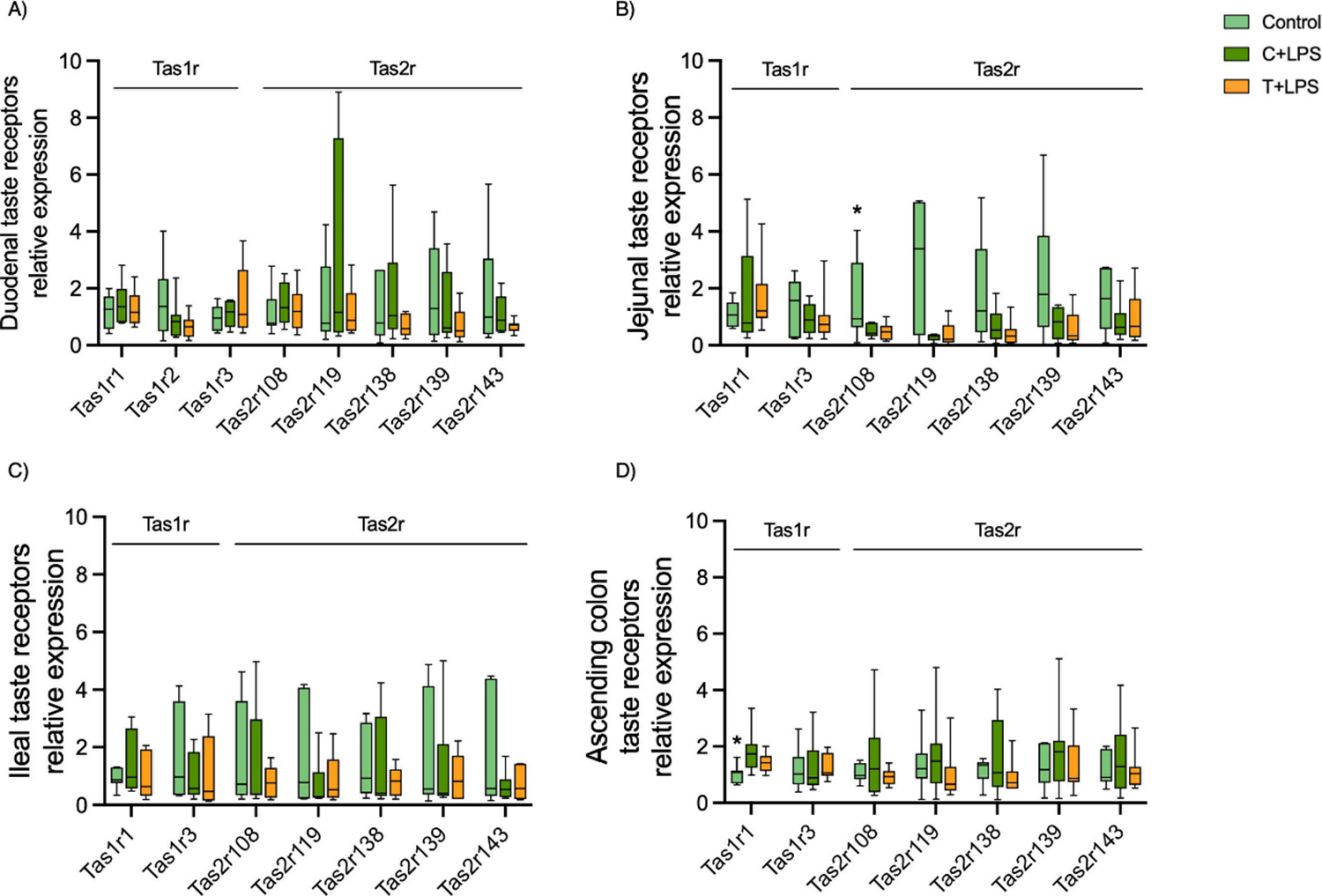
Relative expression of intestinal taste receptors in LPS groups. Animals were treated by 5-days LPS injection (dark green) or by LPS injection plus *Tenebrio molitor* (dark orange), or water as the vehicle (green) for 21 days. The data are presented in a box and whiskers plot. Boxes represent the median and interquartile range, while whiskers extend from the smallest to the largest values, encapsulating the full range of the data. Mann-whitney analysis were used to compare Control and T + LPS with LPS group (*n* = 7–8/group). * indicates *p* < 0.05 compared to LPS group.

We have previously shown that LPS-intestinal-induced inflammatory stimulus caused an inflammatory profile at intestinal and peripheral levels that is in some respects ameliorated by insect consumption.^37^ In this paper, we have investigated the impact of LPS on intestinal taste receptors and explored the effects of insect consumption in this inflammatory scenario. Our results revealed that the injection of LPS had minimal impact on the assessed intestinal taste receptors, with minor significant changes observed. No differences were observed in taste receptor expression in the duodenum (Figure 4A). In the jejunum, the relative expression of Tas2r108 was significantly lower in the LPS group than in the control group (Figure 4B). The expression of taste receptors in the ileum did not change with LPS injection (Figure 4C). In the ascending colon, the relative expression of Tas1r1 increased significantly in the LPS group (Figure 4D). Similarly, *T. molitor* supplementation in the inflammatory model did not change the expression of taste receptors in any part of the intestine compared to that in LPS-treated animals.

The relative expression of Tas1r1 in the descending colon remained unchanged after LPS injection with or without *T. molitor* administration (control: 1.12 ± 0.44; *Tenebrio* + LPS: 1.75 ± 0.70; LPS: 1.68 ± 0.61; *p* > 0.05). Tas1r3 expression in the descending colon also showed no significant changes between groups (control: 1.06 ± 0.37; *Tenebrio* + LPS: 0.97 ± 0.21; LPS: 1.04 ± 0.29; *p* > 0.05).

### Gene Expression of Colonic Taste Receptors Played a Lesser Role As Key Variables for Discriminating between Rats in an Inflammatory State and Those in the Control Group

**Figure 5:**
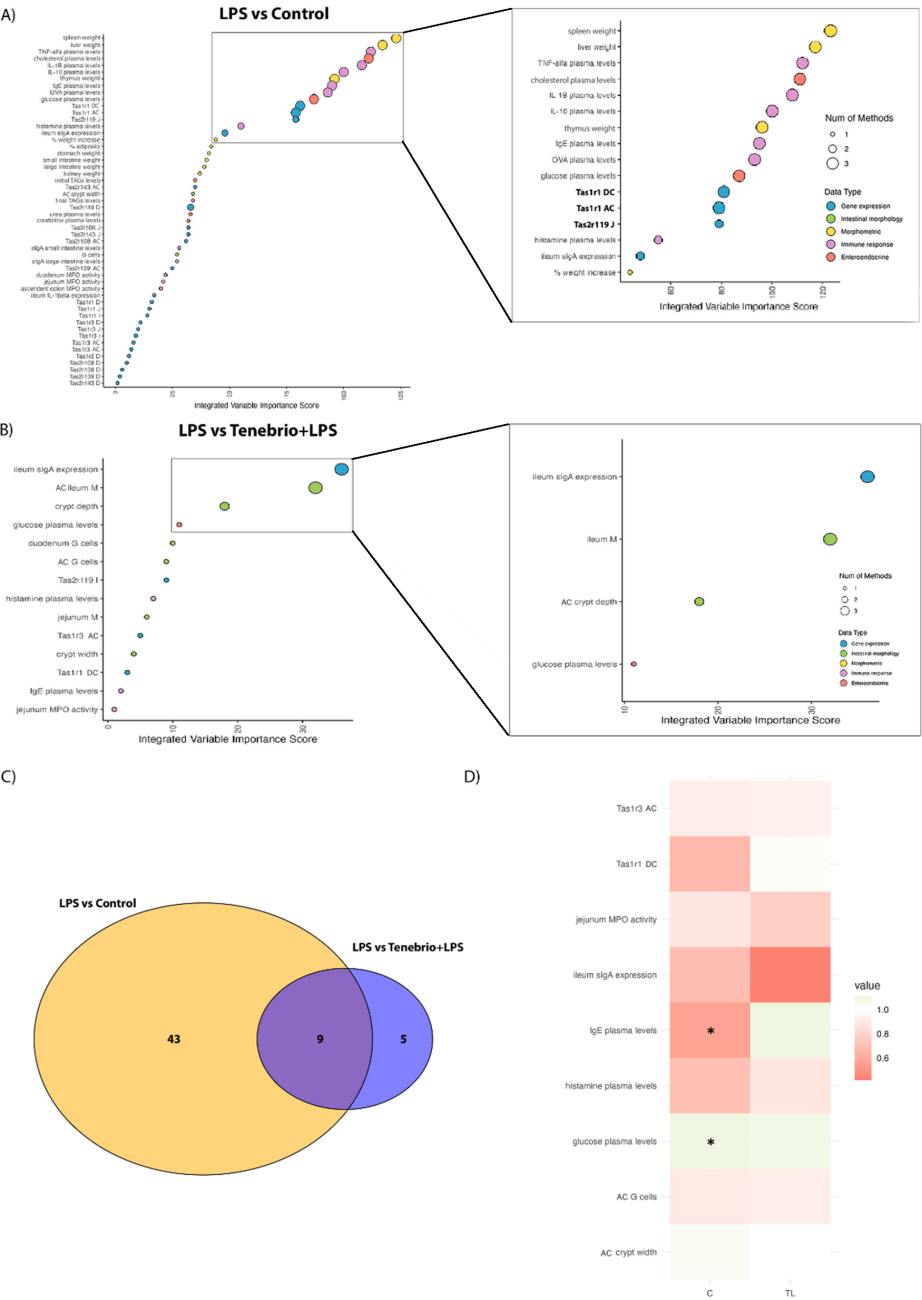
Principal variables distinguishing between the LPS and the control or T + LPS groups. (A and B) integrate the analysis of selected variables using machine learning algorithms, ranking them to distinguish between LPS and C or LPS and T + LPS, respectively. (C) Venn Diagram derived from the integrative analysis. (D) Fold change Heatmap for the nine variables that are common in the two comparisons. Green color indicates the gene expression upregulation or higher levels of the variable, while red signifies downregulation or reduced levels, compared to LPS. * indicates significant differences. Abbreviations utilized include D (duodenum), J (jejunum), I (ileum), AC (ascending colon), DC (descending colon), G (goblet cells), and M (total absorptive area).

Integrative analysis revealed that, under the proinflammatory stimulus, immunological variables and morphological factors were the main parameters for distinguishing between the control group and the LPS-treated group (Figure 5A). However, of the fifty-two selected variables, 18 taste receptors played a crucial role in this differentiation. Most notably, in the subset of most important variables, the relative expression of taste receptor Tas1r1 in the colon and that of Tas2r119 in the jejunum are also particularly influential in distinguishing between the control group and the LPS-treated group. The lesser importance achieved by taste receptors for discriminating between the LPS-treated group and the control group conditioned the importance of these receptors when the effect on *Tenebrio* consumption was analyzed under this inflammatory status. In the integrative analysis of the relationship between the LPS and the *Tenebrio* + LPS groups, fewer variables were selected by the machine learning algorithms (Figure 5B). However, the relative expressions of colonic umami taste receptors Tas1r1 and Tas1r3, along with the ileal taste receptor Tas2r119, were again selected (but only by one of those algorithms).

When we combined the two integrative analyses in the Venn Diagram, nine of the fifty-two variables that differed between the control group and the LPS groups also differed between the LPS groups (Figure 5C). Thus, in this case, the Fold Change heatmap (Figure 5D) represents an increase or decrease of a variable with respect to the LPS group. It clearly shows the role of type-I taste receptors in the descending colon in distinguishing between groups. Specifically, Tas1r3 in the ascending colon exhibits a consistent direction in both the control group and the *Tenebrio* + LPS group, which indicates the potential prevention of LPS-induced alterations. Tas1r1 in the descending colon is also key in separating the LPS group from the control group, where the LPS induces an upregulation of its expression. In this context, insect consumption also increases the relative expression of Tas1r1.

### Interplay between Taste Receptors and Immunological and Metabolic Markers

Integrative analysis earlier identified certain taste receptors with an important role in explaining the effects of an insect-enriched diet in both healthy and inflammatory scenarios. We then conducted Spearman correlation analysis to assess the relationships between biochemical, morphometric, or immunological parameters and taste receptor expression. We ran two separate analyses depending on the situation: the *Tenebrio* and Buffalo groups on the one hand and the LPS-injected groups on the other. We focused especially on taste receptors in the jejunum, ileum, and ascending colon as these were identified as the primary variables by machine learning analysis.

**Figure 6:**
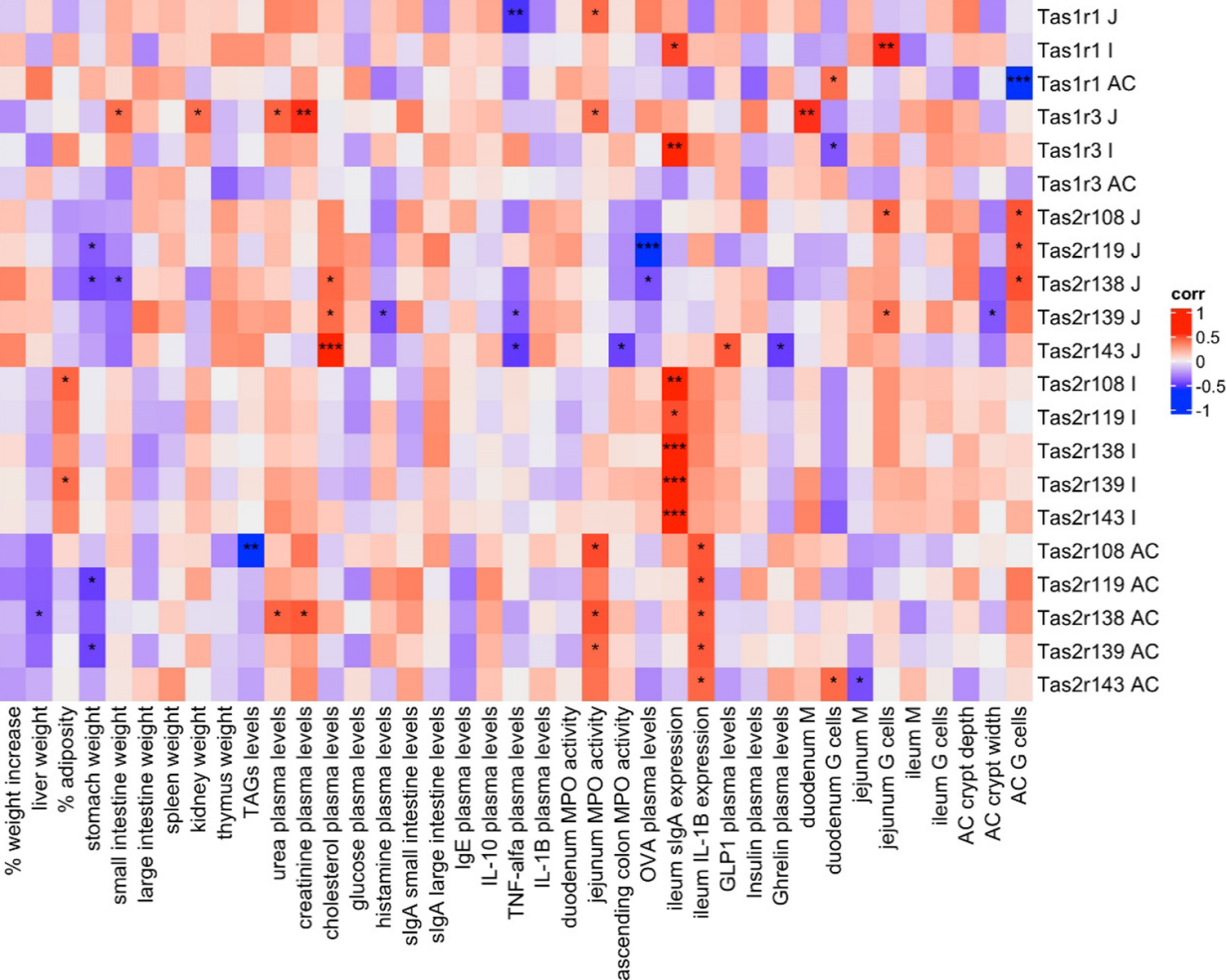
Heatmap of spearman correlations between taste receptor relative expression and the other parameters in *Tenebrio* and Buffalo groups and control. Red color indicates positive correlations while blue color indicates negative correlations. *, ** or *** correlation’s *p*-value <0.05, 0.01, or 0.001, respectively.

In the homeostatic situation, the integrative analysis highlighted Tas1r1 in the ascending colon as an important taste receptor for explaining the interaction of insects with the organism. Figure 6 shows that the expression of Tas1r1 has a strong negative correlation with the percentage of goblet cells also present in the colon. Tas2r119 in the jejunum also had an important role in the integrative analysis in both insect species. We can see that the expression of Tas2r119 exhibited a negative correlation with plasmatic levels of ovalbumin, a parameter associated with the integrity of the intestinal barrier. Moreover, the expression of the ileal taste receptors demonstrates strong positive correlations with those of ileal secretory IgA: specifically, Tas2r138, −139, and −143 (*p* < 0.001).

With regard to the interplay between taste receptor expression and metabolic parameters in this homeostatic condition, plasmatic cholesterol is positively correlated with bitter taste receptors of the jejunum and, in particular, displays a highly significant correlation with Tas2r143 expression. Moreover, this receptor was also positively correlated to GLP-1 and negatively correlated with ghrelin plasmatic levels.

**Figure 7:**
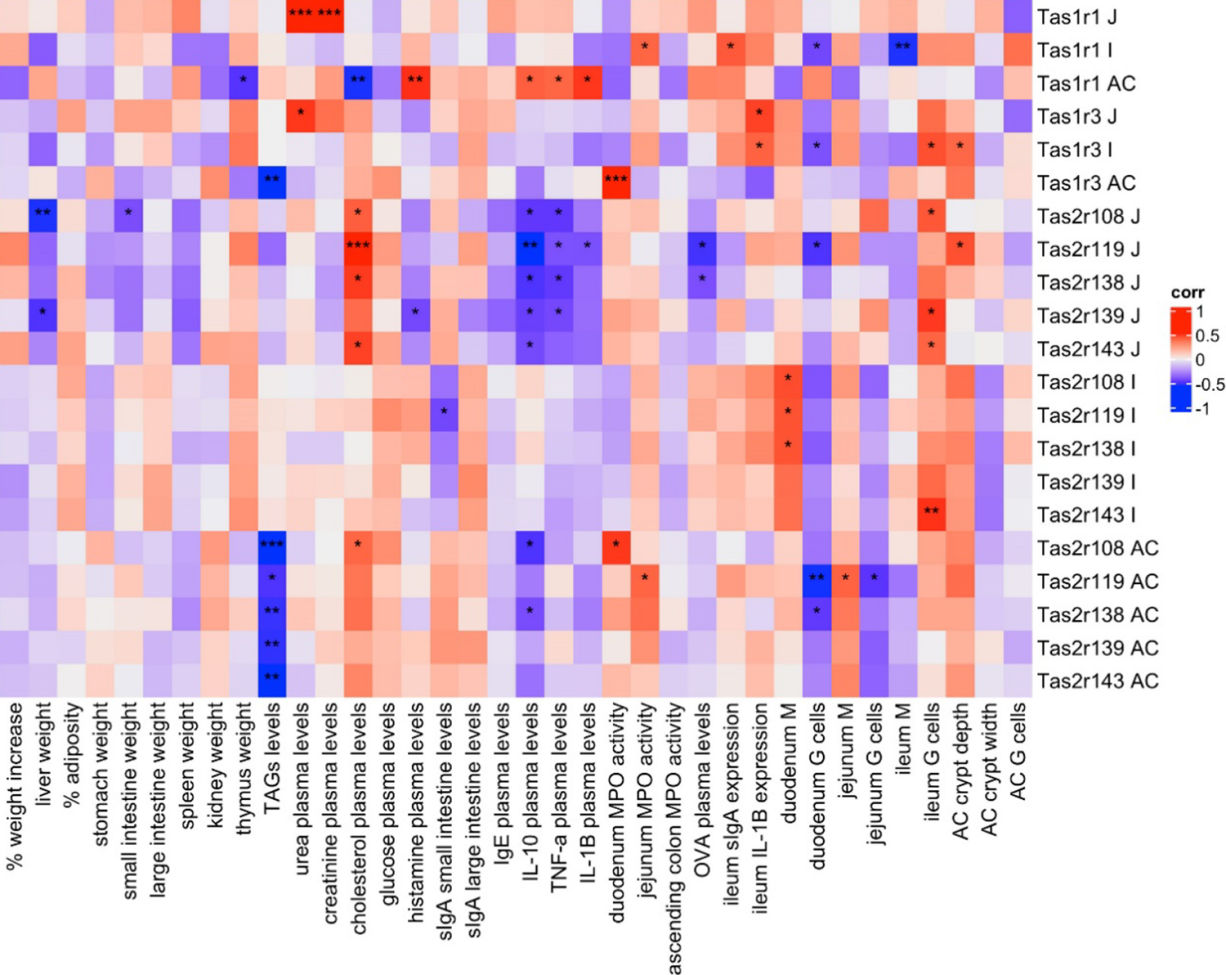
Heatmap of Spearman correlations between taste receptors relative expression and the other measured variables in LPS groups and control. Red color indicates positive correlations while blue color indicates negative correlations. *, ** or *** correlation’s *p*-value <0.05, 0.01, or 0.001, respectively.

In the inflammatory scenario, an earlier integrative analysis also highlighted the modulation of Tas1r1 in the ascending colon. Correlation analysis related this positively with several plasma cytokines and histamine and negatively with plasma cholesterol levels (Figure 7). Moreover, analysis of the Venn diagram and Fold Change heatmap shows that *Tenebrio* treatment prevented the LPS effect on Tas1r3 expression levels in the ascending colon. Observed is a strong positive correlation between these Tas1r3 and duodenum MPO activities and a negative correlation with final plasma TAG levels, the parameter that showed the highest number of correlations (all of them negative) with almost all taste receptors analyzed in the ascending colon. Finally, jejunal bitter taste receptors presented negative correlations with inflammatory cytokines and the intestinal permeability marker (IL-1β, TNF-α, IL-10, and OVA levels, respectively). Interestingly, these negative correlations were significant when correlated with jejunal Tas2r119 expression. Moreover, jejunal bitter taste receptors exhibited positive correlations with final cholesterol levels, whereas negative correlations were observed between plasmatic levels of triglycerides and bitter taste receptors of the ascending colon. Finally, strong positive correlations were observed between jejunal Tas1r1 expression and creatinine and urea plasmatic levels (*p* < 0.001).

## Discussion

The present study reveals, for the first time, the modulation of umami and bitter taste receptor expressions with chronic protein supplementation in female rats. The integrative analysis, which considers multiple variables simultaneously and provides a more holistic perspective of the data than single-level analysis,^44^ identified ascending colonic Tas1r1 expression, along with Tas1r3 and some bitter taste receptors spanning from the jejunum to the colon, as crucial variable distinguishing between the control and the insect-supplemented groups. Additionally, taste receptor expressions significantly correlated with inflammatory and metabolic parameters under both healthy and inflammatory conditions.

First, in a healthy situation, the colonic expression of Tas1r1 and Tas1r3 was upregulated upon supplementation with both Buffalo and *T. molitor*, suggesting taste receptor gene expression can be modulated by insect-derived components in the diet. These findings align with numerous studies in humans and rodents suggesting that chronic exposure to certain dietary compounds, including taste receptor agonists, may lead to changes in mRNA expression of these receptors.^45−47^ Umami taste is often associated with the presence of L-amino acids, especially glutamate and aspartate. Insects, known for their high levels of amino acids, including glutamic acid, serve as an excellent source of umami receptor ligands. In fact, glutamate and aspartate are the most abundant amino acids in the raw Buffalo flour used in this study.^48^ Regarding amino acid consumption, some authors have shown that dietary glutamate increases the expression of Tas1r1 and Tas1r3 in the stomach and jejunum of piglets, along with other glutamate signaling receptors.^49^ Moreover, insect protein is also rich in branched-chain amino acids (isoleucine, leucine, and valine), which have also been described to upregulate umami receptor expression and protein in porcine jejunum.^50^ Therefore, considering all of these factors, increased umami taste receptor expression may be a response to the levels of amino acids in the diet reaching the gastrointestinal tract.

Interestingly, some authors have shown that, in humans and other higher animals, the intestinal secretion of CCK, a satiety hormone, can occur through the Tas1r1/Tas1r3 activation, while others have also suggested GLP-1 secretion after amino acid activation of this receptor.^49,51^ In line with this, previous findings from our research team have demonstrated an increase in plasma GLP-1 levels in rats after insect supplementation.^33^ Moreover, the integrative analysis performed in this study revealed that GLP-1 is another key variable in distinguishing the animal groups that received insect supplementation. Taken together, these results provide more evidence of a possible relationship between umami receptors and hormone secretion in the intestine.

Furthermore, the bitter taste receptors selected by machine learning algorithms were mainly located in the jejunum, ileum, and ascending colon. Among these, jejunal Tas2r119 emerged as a key variable that facilitates the differentiation between the insect-supplemented groups and the control group. These results are also consistent with previous research that described the interaction of certain peptides and amino acids with human bitter taste receptors,^12^ positioning Tas2r119 as particularly susceptible to insect protein consumption. Notably, our findings revealed a significant modulation of both umami and bitter taste receptors in the ascending colon, particularly evident in the colonic Tas1r1 upregulation after insect supplementation and in the Buffalo-supplemented group, which showed significantly lower expression levels of Tas2r119 and Tas2r138. These findings may indicate a specific modulation of taste receptors in the colon that is potentially influenced by the microbiota. The intestinal microbiome has been shown to interact with taste receptors, impacting their expression and function.^52,53^ Additionally, research by Borrelli et al. highlights the potential contribution of insects to this modulation,^54^ as they can serve as sources of short-chain fatty acids (SCFA) known to influence taste receptor expression. SCFA-treated organoids exhibited upregulation of umami gene expression, suggesting a multifaceted mechanism involving not only amino acid composition but also microbial-derived metabolites in taste receptor modulation within the colon.^55^ Moreover, recent studies have even reported a microbial-dependent regulation of Tas2r in mice subjected to a long-term high-fat diet,^56^ further supporting the hypothesis that changes in microbiota composition due to dietary factors may influence taste receptor expression.

The influence of this taste receptor expression modulation on intestinal function and overall organism responses remains unclear. To gain further insights into the potential implications of the expression changes, we conducted a correlation study between the expression of taste receptors and the other evaluated variables. The correlation findings between the expression of colonic Tas1r1 and the percentage of goblet cells in the colon suggested a potential interplay with the colonic mucosal environment. Goblet cells, specialized in producing mucus, play a crucial role in maintaining intestinal health and initiating immunological responses,^57^ reinforcing the idea of Tas1r1 involvement in immune function.^58^ Moreover, this connection between goblet cells and Tas1r1 may be produced by the presence of this receptor in colonic tuft cells, which are involved in the immune response that can activate goblet cells.^59,60^ However, an additional explanation for this negative correlation could be attributed to the influence of insect compounds on the composition of intestinal cells. Several studies suggested that dietary components like nondigestible carbohydrates and polyphenols can promote L-cell differentiation.^61−63^ Therefore, the specific differentiation of those cells expressing Tas1r1, such as L-cells or tuft cells, could logically be related to a decrease in the percentage of goblet cells. Additionally, the positive correlation between the expression of ileal taste receptors and ileal secretory IgA is intriguing and emphasizes the role of taste receptors in the intestinal immune response.^64^ IgA acts as the first barrier on mucosa surfaces against infectious microorganisms and toxins. Similarly, antimicrobial peptides, which may be secreted by the stimulation of Tas2r,^18^ serve as immunomodulators in this context and may impact secretory IgA. These findings collectively set the basis for further research on the specific role of taste receptors in the context of immune response in both healthy and disease situations.

Furthermore, correlation analysis in the two conditions analyzed (healthy and inflammatory-induced animals) revealed a negative relation between the mRNA expression of Tas2r119 and intestinal permeability, also suggesting that this receptor is involved in intestinal function and barrier integrity. This association between bitter taste receptors and permeability has been explored in the context of pulmonary endothelium, where bitter taste agonists demonstrated a reduction in the LPS-induced permeability of the pulmonary endothelium in vitro.^65^

In this study, we also aimed to analyze the effect of protein supplementation in an inflammatory model. The obtained results indicated that the impact of insect consumption under LPS-induced inflammation was more closely associated with other intestinal parameters, such as inflammation or morphometry of the intestine, rather than with the expression of taste receptors. Despite the nondecisive role of taste receptors in differentiating between insect consumption and control groups under inflammatory conditions, the LPS injection did impact the intestinal expression of taste receptors. This model, which is characterized by the production of a spectrum of cytokines that results in systemic inflammation, particularly manifests an altered physical barrier and a proinflammatory intestinal environment in the small intestine.^40,66^ In this inflammatory context, taste receptors may be affected and potentially contribute to the immune response, thus creating a feedback loop.^67^ Machine learning algorithms identified 18 taste receptors that distinguish between the LPS group and the control group. Notably, colonic umami or umami-sweet taste receptors, as well as jejunal Tas2r119, again emerged as the primary contributors to group differentiation. Our univariate analysis showed that the gene expression of Tas2r either did not change or was significantly decreased in certain parts of the intestines of rats with LPS-induced inflammation. Similarly, another study in mice reported an inhibition of taste bud cell renewal after the intraperitoneal injection of LPS.^68^ In contrast, a previous study that investigated the expression of some type-II taste receptors in taste buds found that injecting LPS stimulated Tas2rs expression also in mice.^69^ Even with these contradictory findings, our results help to establish a connection between taste receptors and immune disturbance, as has previously been described for both Tas1r and Tas2r in humans and rodents.^64,70^

Our correlation results (particularly in LPS-injected rats but also in healthy ones) showed negative correlations between plasmatic TNF-α, IL-1β, and IL-10 levels, and bitter taste receptors (especially Tas2r119) in the jejunum as well as a positive correlation with colonic Tas1r1. Similarly, a study by Reynolds et al. reported an increase in Tas1r1 expression in conjunction with an immune response.^22^ Moreover, our study demonstrates that colonic Tas1r3 is also a key discriminator between (i) LPS-injected rats and those in the control group and (ii) rats injected with LPS alone and those also administered *Tenebrio*, showing upregulation specifically in the LPS group. This is in line with the results of the study by Shon et al., which suggest that Tas1r3 is a mediator of intestinal inflammation in mice.^4^

Finally, interesting correlations are observed in the LPS-treated groups with regard to the biochemical variables analyzed. While final triglycerides are negatively correlated with ascending colon taste receptors, cholesterol levels are positively correlated with jejunal bitter ones. In this context, previous studies in humans and rodents have reported a possible relationship between intestinal bitter taste receptors and lipid metabolism.^71^ In addition, recent research has added a new dimension to this relationship by showing that cholesterol acts as an agonist for bitter taste receptors, modulating their function.^72,73^ Together, these findings highlight the intricate interplay between lipid metabolism and taste receptor signaling pathways, potentially opening up new avenues for understanding and treating metabolic diseases. Furthermore, creatinine and urea plasmatic levels correlate with jejunal umami taste receptors, suggesting possible links between umami taste perception and amino acid metabolism. Dietary glutamate and aspartate are metabolized during intestinal transport to various products, including urea metabolites.^74,75^ In this regard, previous findings from our group showed that insect supplementation in rats ameliorates LPS-altered urea levels in plasma,^37^ reinforcing the link between L-amino acid levels reaching the jejunum and plasma urea levels. Thus, the correlations observed in our study raise the possibility that the activation of umami intestinal taste receptors could play a role in the regulation of nitrogen metabolism. All of this novel evidence poses questions about the potential systemic effects of taste receptor modulation beyond the digestive system and suggests that taste receptors can be explored as potential targets for treating disturbances.

One limitation of our study is that while we observed modifications in gene expression, these changes may not necessarily translate directly to alterations in protein levels. This is mainly related to the level of expression of these receptors and the scarce availability of technical tools to run this screening and quantify them as transmembrane proteins. Additionally, our analysis was conducted on whole intestinal tissue without specific cell-level resolution. However, it is essential to note that our study serves as an initial screening, revealing the modulation of taste receptor expression by different treatments. This preliminary investigation provides a foundation for future research to delve deeper into the most significant receptors and their specific cellular locations within the intestinal tract as well as to elucidate the underlying mechanisms involved. Further studies focusing on these aspects are crucial for a comprehensive understanding of how insect protein supplementation influences taste receptor expression and function in the gut. Additionally, identifying the specific constituents of insect flour, such as insect-specific 5-ribonucleotides, amino acids, or peptides, responsible for these effects is crucial.

In conclusion, this study demonstrates the modulation of taste receptors after various interventions in rat models. Our data postulate the expression of Tas1r1 in the ascending colon as a relevant taste receptor regulated by insect consumption. The consistency of our results across multiple analytical methods strengthens the validity of our findings, which suggest that insect supplementation or induced inflammation modified taste receptor expression, particularly colonic umami taste receptors, that could involve changes in intestinal function and systemic health. Moreover, the intricate network of correlations between taste receptor expression and several physiological parameters, such as various morphological, biochemical, and inflammatory parameters, emphasizes the complexity of interactions within and beyond the gastrointestinal system. Hence, our findings can contribute to a better understanding of the complex mechanisms regulating diet-health interactions, facilitating the development of targeted nutritional interventions toward enhancing intestinal health and overall well-being. Nevertheless, further research is needed to fully elucidate the direct relationship among intestinal taste receptors, gut functions, and overall health.
